# The effectiveness of gaming interventions for depression and anxiety in young people: systematic review and meta-analysis

**DOI:** 10.1192/bjo.2021.1078

**Published:** 2022-01-07

**Authors:** Christopher Townsend, Clara Humpston, Jack Rogers, Victoria Goodyear, Anna Lavis, Maria Michail

**Affiliations:** Institute for Mental Health, School of Psychology, University of Birmingham, UK; Institute for Mental Health, School of Psychology, University of Birmingham, UK; Institute for Mental Health, School of Psychology, University of Birmingham, UK; Institute for Mental Health, School of Psychology, University of Birmingham, UK; and School of Sport, Exercise and Rehabilitation Sciences, University of Birmingham, UK; Institute for Mental Health, School of Psychology, University of Birmingham, UK; and Institute of Applied Health Research, College of Medical and Dental Sciences, UK; Institute for Mental Health, School of Psychology, University of Birmingham, UK

**Keywords:** Gaming interventions, serious games, depression, anxiety, youth mental health

## Abstract

**Background:**

Recent research has investigated the use of serious games as a form of therapeutic intervention for depression and anxiety in young people.

**Aims:**

To conduct a systematic review and meta-analysis into the effectiveness of gaming interventions for treating either depression or anxiety in individuals aged 12–25 years.

**Method:**

An electronic search was conducted on the 30 March 2020, using PsycINFO, ISI Web of Science Core Collection, Medline and EMBASE databases. Standardised effect sizes (Hedge's *g*) were calculated for between-participant comparisons between experimental (therapeutic intervention) and control conditions, and within-participant comparisons between pre- and post-intervention time points for repeated measures designs.

**Results:**

Twelve studies (seven randomised controlled trials (RCTs) and five non-randomised studies) were included. For RCTs, there was a statistically significant and robust effect (*g* = −0.54, 95% CI −1.00 to −0.08) favouring the therapeutic intervention when treating youth depression. For non-RCTs, using a repeated measures design, the overall effect was also strong (*g* = −0.75, 95% CI −1.64 to 0.14) favouring therapeutic intervention, but this was not statistically significant. Interestingly, we found no statistically significant effect for treating youth anxiety.

**Conclusions:**

There is preliminary evidence to suggest that gaming interventions are an effective treatment for youth depression, but not anxiety. Further research is warranted to establish the utility, acceptability and effectiveness of gaming interventions in treating mental health problems in young people.

Depression and anxiety are among the most commonly reported mental health problems in children and young people.^1^ Costing UK society £70–£100 billion/year, early-life disorders such as depression and anxiety are linked to poor psychosocial outcomes, including lower educational attainment, a 25–30% increase in the likelihood of dropping out of school, unemployment, lower socioeconomic status and reduced social support.^2,3^ Approximately 8.1% of 5- to 19-year-olds in the UK present with depression.^4^ The overall prevalence of depression in young people aged 15–24 years is estimated to be 11%; a rate that has been steadily increasing over the past decade.^5^ Similarly, recent findings from the World Health Organization's World Mental Health International College Student Project indicate major depressive disorder and generalised anxiety disorder are the two most common disorders experienced by first-year college students, with a median age at onset of 14.3 and 14.6 years, respectively.^6^

Clinical guidelines in the UK recommend psychological therapy (e.g. cognitive–behavioural therapy (CBT) and family therapy) as the first-line treatment for both depression and anxiety in children and young people.^7^ However, problems with access to mental health services and/or long waiting times, stigma associated with receiving formal mental health support and a lack of mental health knowledge present significant challenges to help-seeking.^8^ Given the high socioeconomic burden and personal costs of both depression and anxiety in adolescence, and the often low accessibility of psychological treatments such as CBT, the development of new pragmatic, effective and scalable methods for treating depression and anxiety in young people is a high priority, for the benefit of those who seek mental health treatment and those who provide it.

## Gaming use in mental health

Through its popularity, accessibility and relevance in many young people's lives, gaming remains a promising treatment avenue for mental health problems.^9,10^ In the UK, 81% of 12- to 15-year-olds play games for over 11 hours per week on various devices, such as consoles, smartphones and tablets.^11^ Children and young people engage with various types of games, including single and multiplayer games, competitive games, strategy games, e-sports, exergames and virtual reality.^12^ Boys are more likely to play games than girls online;^13^ however, the proportion of girls who play games increased from 38% in 2018 to 48% in 2019, whereas the proportion of boys who play games online has remained stable at 71%.^11^

Gaming interventions for mental health vary in methodological approach and have been categorised in the following ways: exergames (games that involve physical exertion as a form of exercise), virtual reality (games that use computer-generated simulations of a three-dimensional environment, allowing user interaction), CBT-based games (games utilising/integrating the principles of cognitive therapy), entertainment games (games aimed at giving the player a sense of pleasure, which could help with motivation and learning), biofeedback (electronic monitoring of a physiological function that is fed back to the individual, usually as a mini-game, for better voluntary control of the said physiological function) and cognitive training games (games designed to improve certain cognitive functions, such as memory).^14^ Most gaming interventions for mental health fall under the broader category of ‘serious games’.^15^ In contrast to most commercial games that are designed for the purpose of entertainment, serious games are designed for treatment, using gaming principles to support mental health problems.^16^ Gaming interventions for mental health often feature a system of reward, are interactive and/or competitive and are designed to be ‘fun’.^17^

Because of the emergent nature of gaming interventions, their outcomes among populations have been highly heterogeneous, with best practices for development and evaluation yet to be defined.^18^ However, early research on the implications of gaming for mental health and gaming interventions for treatment purposes has found that gaming can help address key barriers, such as accessibility of treatment, long waiting times and lack of motivation to participate in and engage with the delivery of treatment.^19,20^ For example, gaming can almost always be accessible as it is independent of time and location, and the global prevalence of digital content allows for easy distribution to masses of people largely irrespective of circumstance, giving gaming an advantage over traditional treatments.^21^ Granic et al^20^ also detail how therapy-based video games can address the gap between knowledge and behaviour that exists in CBT approaches, as there is often a disconnect between ‘what youth actually know and what they do in their everyday lives’ (p. 75). The authors explain how gaming interventions engage players in an immersive emotional experience, providing a practice space for new regulatory skills, such as problem-solving, to become automatised through exposure to authentic conflicts and exercises, leading to generalisation outside of the game environment.

Gaming interventions differ from other evidence-based technological approaches such as computerised cognitive–behavioural therapy (CCBT), as gaming interventions emphasise interactive delivery. CCBT interventions for depression and anxiety use a combination of multimedia formats to support delivery and management, including videos, images and cartoons,^22,23^ but lack the interactive elements that video gaming possesses, as seen in SPARX.^24^ SPARX is an interactive fantasy game, aimed at adolescents aged 12–19 years, and is designed to deliver CBT for the treatment of clinically significant depression. In SPARX, users choose an avatar to undertake challenges within the virtual world, as set by a ‘guide’ who gauges user mood, provides education and sets real-life challenges. The effectiveness of the SPARX intervention was tested against a treatment-as-usual group, consisting primarily of face-to-face counselling. Findings for SPARX showed clinically significant improvements in depression symptoms in children seeking help from primary care settings, as measured by reductions on the Children's Depression Rating Scale – Revised at 2 months post-intervention.

More broadly, outcomes of early gaming interventions for the treatment of mental health symptoms, including depression, have reinforced gaming as an effective treatment approach. A meta-analysis exploring game-based interventions for depression therapy found a moderate effect size (*d* = –0.47), but with high heterogeneity at post-treatment for their subgroup analysis on adolescents up to 18 years old.^25^ Similarly, a systematic review investigating the effectiveness of serious games in treating or preventing depression in young people aged 9–25 years generated mostly positive results, with significant improvements in depression scores across studies, except for one.^15^ Moreover, subgroup analyses revealed that serious games exhibited a moderate effect (*g* = 0.55, *P* < 0.001) on improvements in mental health-related symptoms (including depression-related symptoms, cognitive decline symptoms and recognition ability in autism spectrum disorder), favouring serious games over controls with no intervention in randomised controlled trials (RCTs) of young people up to the age of 18 years.^16^ Although providing an insightful look at the effects of gaming interventions, given the infancy of the field, these findings suffer from limited scope and high heterogeneity, with caution needed in attempting to generalise results.^25^

The evidence base on the effectiveness of gaming interventions for anxiety in young people is even less robust than that for depression, after no serious games primarily targeting anxiety were documented in an existing review.^16^ However, since this finding, anxiety-related gaming interventions have begun to emerge, such as MindLight;^26,27^ this trains young people to cope with anxiety by immersing them in a playful, horror-themed world, implementing neurofeedback techniques to alter the game's environments. When compared with a non-gaming CBT-based program, MindLight^26^ was found to be as effective as CBT in reducing anxiety symptoms, with a larger decrease in symptoms for MindLight between post-test and 6-month follow-up.

## Present study

Gaming and its effectiveness in the treatment of depression and anxiety among children and young people is a rapidly evolving field of research. Gaming interventions for the treatment and prevention of depression have garnered improvements in reported levels of depression, yet reviews have noted the infancy of the field and have called for more research to be conducted and developed, as evidence has been very limited.^15,25^ Most reviews have included research published before 2014.^15,25^ Although these reviews provide important evidence on the potential of gaming interventions for depression, these findings may quickly become irrelevant because of the exponential growth in gaming use and access,^11^ and its materialisation in mental health treatment.^14^ Notably, there have been developments in the areas of immersive gaming experiences, and the communicative dimensions of gaming. Similarly, as seen with findings from Lau et al,^16^ anxiety-based gaming interventions are not contemporaneous with research conducted during this time. Thus, a more recent examination of advances within this rapidly developing field is needed to evaluate the outcomes from emergent interventions.

This systematic review and meta-analysis aims to address the aforementioned gaps in research by evaluating the effectiveness of gaming interventions in treating symptoms of depression and/or anxiety in young people aged 12–25 years. The findings from this review can be used to inform the development of robust guidance on the design of gaming interventions for the treatment of mental health problems, to increase their potential to elicit positive changes in outcomes related to anxiety and depression in children and young people.

## Method

### Protocol and registration

This review was conducted and is reported in accordance with Preferred Reporting Items for Systematic Reviews and Meta-Analyses (PRISMA) guidelines.^28^ An *a priori* protocol was registered on International Prospective Register of Systematic Reviews (PROSPERO; identifier CRD42020172243). As this is a systematic review and meta-analysis of published literature, ethical approval was not sought.

### Search strategy

The search strategy was developed with an academic skills specialist at the University of Birmingham, UK. An electronic search was conducted on the 30 March 2020, using PsycINFO, ISI Web of Science Core Collection, Medline and EMBASE databases. The search terms were as follows: Games or Game-based adj1 (treatment or therap* or intervention) or computer* or video* or serious or digital* or web or internet or online or therap* or electronic or virtual adj1 (game* or gaming) in combination with Depress* or Anxi* or Stress or Mood or ‘Mental health’ or ‘Mental disorder’ or Psychotherap* in combination with Child* or adolesc* or ‘young people’ or teen* or kid* or pupils or youth or juvenile or ‘young adult*’ or ‘young person’ or minor*. The search was limited to peer-reviewed papers in the English language, with no date restrictions. Two of the authors (C.T. and C.H.) conducted the literature search independently. Reference lists of eligible papers were also screened for additional articles.

### Eligibility criteria

The eligibility of studies was confirmed according to their adherence to the following inclusion criteria: (a) studies must be published in peer-reviewed journals, in the English language and have an available full-text version; (b) studies must provide data for populations between the ages of 12–25 years, but a mean population age falling within this restriction is deemed eligible (this age range was chosen as the target population as the age at onset is most prevalent across these ages, and three-quarters of mental health problems are established by 24 years of age);^29^ (c) studies must implement a gaming intervention that has been specifically designed for the purpose of therapeutic use in treatment for depression and anxiety; (d) the intervention would be considered to be a ‘gaming intervention’ if it (i) has a system of reward, incentive and/or objective; (ii) is interactive and/or competitive and (iii) was designed for recreational use (i.e. designed to be fun) as defined by Primack et al;^17^ and (e) primary outcomes must be depression and/or anxiety as assessed by a validated outcome measure such as the Children's Depression Rating Scale – Revised (CDRS-R)^30^ or diagnostic interview, self-reported or clinician administered at both baseline and post-intervention. Studies could be RCTs or non-RCTs, including between-participant designs and repeated measures (within-participant) designs. Online therapies such as CCBT were excluded, unless they had built-in interactive gaming components and environments that facilitated the therapy/intervention, aligning with our definitions of a game/serious game. Study participants suffering from a physical health condition (i.e. sample taken from paediatric hospital wards) or who had an intellectual disability were excluded.

Assessment of study eligibility from title and abstract screening through to final inclusion was independently rated by the same two authors who carried out the search for papers. If agreement regarding the eligibility of an article could not be met through discussion, a third reviewer (M.M.) was invited to review.

### Data management and extraction

The screening process and subsequent management of eligible papers were done with Zotero for Windows (version 5.0.96, George Mason University, USA; https://www.zotero.org/). Collections of exported search results at each screening interval were created and made to record each stage of screening. Both authors responsible for the literature search and screening process managed their own independent Zotero libraries, and would then share their libraries to determine agreement on eligibility.

Data from each full-text paper eligible for inclusion were extracted using the Cochrane Effective Practice and Organisation of Care (EPOC) data collection form.^31^ Each of the included studies had information extracted in accordance with the EPOC tool. The following data were extracted: study population (mean age, age range, gender, ethnicity, setting), study design, sample size, experimental group (intervention, description, delivery), comparison group (description, delivery, active control/waitlist control/different gaming intervention/non-gaming intervention), primary outcome measure and any secondary outcome measure (at baseline, post-intervention, follow-ups), study attrition and time points reported of each assessment of the primary/secondary outcome. Data were extracted by two authors (C.T. and C.H.) independently, and both convened upon completion to verify and review extractions.

### Quality assessment

Individual studies were assessed for their risk of bias and quality of design, by two authors (CT + CH), using version 2 of the Cochrane Collaboration risk-of-bias tool for RCTs (RoB 2).^32^ This tool provides a summary assessment of the protocol for each study, with judgements on the randomisation process, missing/incomplete data, deviation from intended intervention, blinding of outcome and selective outcome reporting. Each of the criteria can have an assessment of ‘low risk’, ‘some concerns’ or ‘high risk’, formulating an overall bias judgement. Meanwhile, non-RCTs were assessed with the Risk of Bias in Non-Randomized Studies – of Interventions (ROBINS-I) assessment tool.^33^ Determining bias with the ROBINS-I is predicated on the following criteria: bias owing to confounding, selection of participants, classification of interventions, deviations from intended interventions, missing data, bias in measurement of outcomes and selection bias of reporting result. Outcomes of study designs would ultimately be assessed as having a low risk, moderate risk or high risk of bias.

### Statistical analysis

Statistical analysis was performed with Review Manager version 5.4 software (RevMan 5 for Windows, Cochrane Collaboration, London, UK; see https://training.cochrane.org/online-learning/core-software-cochrane-reviews/revman/revman-5-download).^34^ The meta-analysis implemented a random effects model as between-study heterogeneity was expected to be high. Depression and anxiety outcomes of the meta-analysis were measured through mean and s.d. The standardised mean differences (SMD) statistic summarised the results with 95% confidence intervals, and effects were weighted with the inverse-variance method. Results of the analysis represented the effectiveness of the experimental intervention, with inferences calculated through *Z*-statistics with corresponding *P-*value, presented through forest plots.

Heterogeneity of effect sizes was assessed with the chi-squared value (*χ*²) and *I*^2^ index,^35^ which were derived from Cochrane's *Q*-statistic,^36^ where low (25%), medium (50%) and high (75%) levels of heterogeneity were determined.^37^ Funnel plots also indexed whether there was evidence of publication bias in the analysis, where asymmetry of the funnel plot would indicate greater publication bias (Supplementary Figure 1 available at https://doi.org/10.1192/bjo.2021.1078).

### Analytic strategy

The meta-analysis was separated by distinctions between RCT and non-RCT studies. Non-RCT studies were subdivided and separated further by distinguishing between studies based on between-participant comparison designs versus repeated measures designs. Depression effect sizes and anxiety effect sizes were analysed separately.

Depression and/or anxiety symptoms were used as the primary outcomes. In cases where studies used multiple measures of depression and/or anxiety, only the primary outcome measure as stipulated by the study was extracted and used in the analysis. Effect sizes of RCTs and non-RCTs were calculated as Hedge's *g*, as this corrects Cohen's *d* effect sizes for small sample sizes. RCT and non-RCT group comparison studies assessed effects through the mean changes from baseline assessment to the initial time point post-intervention assessment, whereas effect sizes for non-RCT repeated measures studies were assessed by the mean change from pre- to post-intervention. As pre- versus post-intervention scores for repeated measures are statistically dependent,^38^ a standard within-study correlation coefficient of 0.5 was implemented into each mean change, in accordance with other meta-analysis,^39^ as these correlations were not known.

Effect of the gaming interventions for RCTs and non-RCT group comparisons were determined by the differences in effect sizes between the therapeutic gaming intervention group and control groups. In instances where trials used more than one control group, the waitlist control condition was used in the analysis, as opposed to ‘active’ control conditions.

## Results

**Fig. 1:**
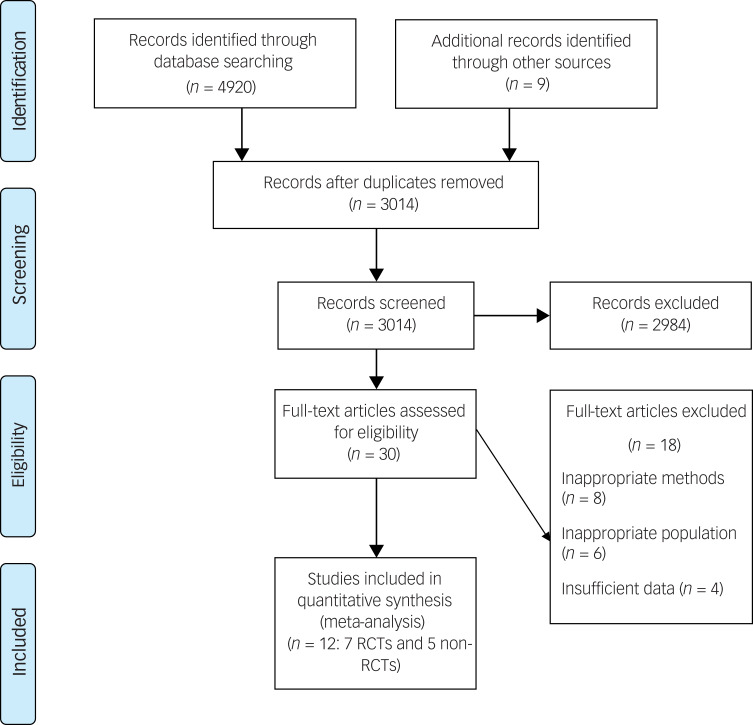
Preferred Reporting Items for Systematic Reviews and Meta-Analyses flowchart for study selection. RCT, randomised controlled trial.

The results of the systematic search are presented in Figure 1. Overall, the searches yielded 4920 results; after duplicates were removed, 3014 records remained for title and abstract screening. Seven RCTs and five non-RCTs that met the inclusion criteria were included in the final analysis (*N* = 12).

### Study characteristics

Table 1 summarise the characteristics of included studies. In total, 844 participants were included in the RCTs, with their mean age ranging between 13.02 and 22.34 years. One RCT was conducted in Romania,^40^ one in the USA,^41^ one in Ireland,^42^ two in New Zealand^24,43^ and two in The Netherlands.^44,45^ For RCTs, SPARX^24^ was the most commonly used active intervention, which focused on learning CBT-based skills to navigate fantasy game-world consisting of seven modules at 20 min each. Three RCTs had more than two arms, usually with one active intervention and several control interventions that may also include a different game as an active control. Four RCTs had more than one follow-up time point varying between 2 and 12 months. Apart from the study by Merry et al,^24^ all RCTs had small-to-moderate sample sizes of no more than 100 per group (range 11–70). There was also significant variability in the choice of measurement for the primary outcome; apart from the CDRS-R (used in four studies), only two studies used the same primary outcome measure,^43,46^ even when the active intervention was the same (e.g. SPARX). The method of delivery for gaming interventions varied between those delivered by teachers and those supervised by trained therapists and researchers, and the duration of treatment was usually in the range of several weeks (e.g. for SPARX, it was delivered as one module per week).

**Table 1:** Characteristics of studies included in the current review

Authors	Year	Total, *N*	Mean age, years (range)	Experimental design	Primary outcome	Outcome measure	Location	Intervention group	Intervention description	Intervention delivery	Intervention group, *n*	Control group	Control group, *n*	Main findings
David et al^40^	2019	96	13.02 (10−16)	RCT	Depression	EATQ-R	Romania	REThink	Progression through seven levels lasting maximum 50 min each	iOS application	48	Waiting list	48	Participants reported reduced depressive mood symptoms following REThink (*d* = 0.84)
Dennis and O'Toole^41^	2014	38	22.34 (17−50)	RCT	Anxiety	STAI	USA	ABMT	Points earned through successful completion of tasks consisting of 16 blocks of 40 trials	iOS application	19	Placebo training	19	Reduced subjective anxiety post-intervention in only the short ABMT condition
Dennis-Tiwary et al^46^	2016	42	20.60 (18−38)	Non-RCT	Anxiety	STAI	USA	ABMT	Points earned through successful completion of tasks consisting of 12 blocks of 40 trials	iOS application	19	Placebo training	23	STAI scores did not reach significance; women reported improved performance on anxiety stress-related task
Fleming et al^43^	2012	30	14.9 (13−16)	RCT	Depression	CDRS-R	New Zealand	SPARX	Learned skills to navigate fantasy game-world consisting of seven modules at 20 min each	Computer program	19	Waiting list	11	SPARX group reported significantly greater reduction in CDRS-R scores compared with waiting list
Knox et al^47^	2011	24	12.88 (9−17)	Non-RCT	Anxiety; depression	MASC; CDI	USA	Freeze-Framer 2.0 and Journey to the Wild Divine	Biofeedback programs where players interact with a fantasy world and face challenges to overcome when navigating	Biofeedback and computer program	12	Waiting list	12	Intervention group reported reduced MASC and CDI scores compared with comparison group
Kuosmanen et al^42^	2017	66	17.60 (15−20)	RCT	Anxiety; depression	GAD-7; SMFQ	Ireland	SPARX-R	Learned skills to navigate fantasy game-world consisting of seven modules at 20 min each (revised version of SPARX)	Computer program	30	No intervention	36	Non-significant treatment effect for both anxiety (*P* = 0.34) and depression (*P* = 0.88)
Lucassen et al^48^	2015	21	16.50 (13−19)	Non-RCT	Depression	CDRS-R	New Zealand	Rainbow SPARX	Learned skills to navigate fantasy game-world consisting of seven modules at 20 min each (revised version of SPARX to adapt program for sexual minority groups)	Computer program	21	−	−	Depressive symptoms decreased significantly post-intervention (*d* = 1.01), which was maintained at 3-month follow-up
Merry et al^24^	2012	374	15.60 (12−19)	RCT	Depression	CDRS-R	New Zealand	SPARX	Learned skills to navigate fantasy game-world consisting of seven modules at 20 min each	Computer program	187	Treatment as usual	187	Participants in intention-to-treat analysis reported mean reduction on CDRS-R scores with advantage to SPARX of 1.60 (*P* = 0.26) over comparison
Poppelaars et al^44^	2016	102	13.35 (11−16)	RCT	Depression	RADS-2	The Netherlands	SPARX	Learned skills to navigate fantasy game-world consisting of seven modules at 20 min each	Computer program	51	No intervention	51	Depressive symptoms decreased across all conditions with all conditions being equally effective
Scholten et al^45^	2016	138	13.27 (11−15)	RCT	Anxiety	SCAS	The Netherlands	Dojo	Biofeedback game in a fantasy world where players interact with characters, and are challenged by them, with emotion states reflected in the game world	Biofeedback and computer program	70	Rayman 2: The Great Escape	68	Anxiety symptoms decreased in both conditions with equal improvement and no significant difference across the conditions
Shandley et al^50^	2010	266	20.50 (18−25)	Non-RCT	Anxiety/ depression	KPDS	Australia	Reach Out Central	Players roleplay as a character who must adjust to new a new environment and are encouraged to perform various ‘real-life’ actions in game to improve mood metre and progress	Online gaming program	266	−	−	Women reported higher mean scores on the KPDS
Shepherd et al^49^	2018	6	14.67 (14−16)	Non-RCT	Depression	CDRS-R	New Zealand	SPARX	Learned skills to navigate fantasy game-world consisting of seven modules at 20 min each	Computer program	6	−	−	SPARX led to significant reduction in CDRS-R scores post-intervention from baseline (*P* = 0.008)

RCT, Randomised Controlled Trials; EATQ-R, Early Adolescent Temperament Questionnaire-Revised; STAI, State-Trait-Anxiety-Inventory; ABMT, Attention Bias Modification Training; CDRS-R, Children's Depression Rating Scale-Revised; SPARX, Smart, Positive, Active, Realistic, X-factor thoughts; MASC, Multidimensional Anxiety Scale for Children; CDI, Children's Depression Inventory; GAD-7, General Anxiety Disorder Assessment, 7-items; SMFQ, Short Mood and Feelings Questionnaire; SPARX-R, Smart, Positive, Active, Realistic, X-factor thoughts-Revised; RADS-2, Reynolds Adolescent Depression Scale, 2nd Edition; SCAS, The Spence Children's Anxiety Scale; KPDS, Kessler Psychological Distress Scale.

In total, 359 participants were included across the five non-RCT studies, with their mean age ranging between 12.88 and 20.60 years. Two non-RCTs were conducted in the USA,^46,47^ two in New Zealand^48,49^ and one was conducted in Australia.^50^ For non-RCTs, two studies adopted a between-participant design (treatment versus control), whereas three used a within-participant (pre- versus post-intervention) design. The study by Shandley et al^50^ had the largest sample size (*N* = 266) compared with the other studies, whose sample size ranged between six and 42. This study was also the only one that used a combined measure of depression and anxiety (the Kessler Psychological Distress Scale). All three within-participant studies had two follow-up time points: one immediately after intervention and a further follow-up at either 2 or 3 months afterward. Again, SPARX was the most commonly reported active intervention (including a modified version, Rainbow SPARX, in which contents specifically adapted for sexual minority youth were added while keeping the same mini-games and characters).^48^

### Risk-of-bias assessments

All RCTs, except for Kuosmanen et al^42^ and Poppelaars et al,^44^ were deemed of low risk based on the RoB 2 tool (see Supplementary Figure 2); those two studies were rated as having ‘some concerns’, and the source of potential bias came from missing data at follow-up (attrition bias). All non-RCTs were rated as moderate risk (the second lowest ranking) according to the ROBINS-I tool (Supplementary Figure 3), given that none of these was considered to be of the same robustness or quality as an RCT as outlined by the Cochrane guidelines. With Shandley et al^50^ as the only exception, all non-RCTs had potential biases with participant selection; however, both Shandley et al^50^ and Lucassen et al^48^ reported issues with missing data. All non-RCTs were rated as having suffered potential biases in the selection of reported results.

### Baseline comparisons

**Fig. 2:**
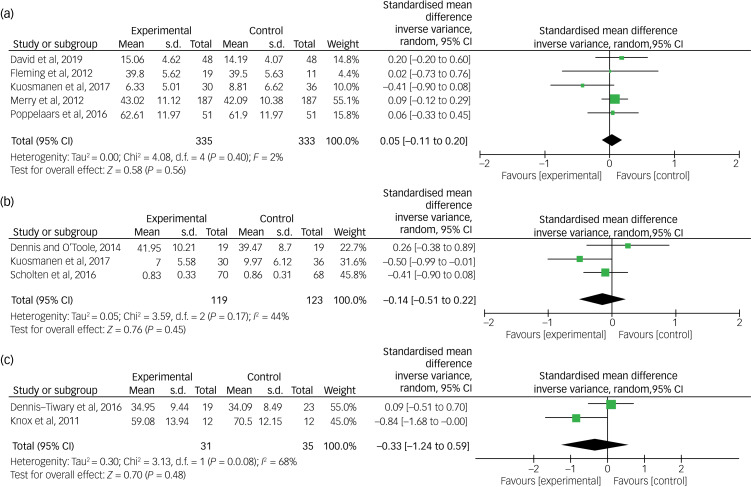
Baseline comparisons for RCTs and non-RCTs. RCT, randomised controlled trial.

Figure 2 shows that there were no significant differences between active intervention and control groups pre-intervention. This was found to be exactly the case for RCTs on depression (Fig. 2(a)) and on anxiety (Fig. 2(b)), as well as non-RCTs that used a between-participant design (Fig. 2(c)), with no statistically significant differences favouring either the experimental or the control condition. Heterogeneity for RCTs treating depression was extremely low at baseline (*I^2^* = 2%), whereas heterogeneity for RCTs targeting anxiety was much higher (*I^2^* = 44%), and heterogeneity for non-RCTs at baseline was even higher (*I^2^* = 68%).

### Effects of gaming interventions on depression

**Fig. 3:**
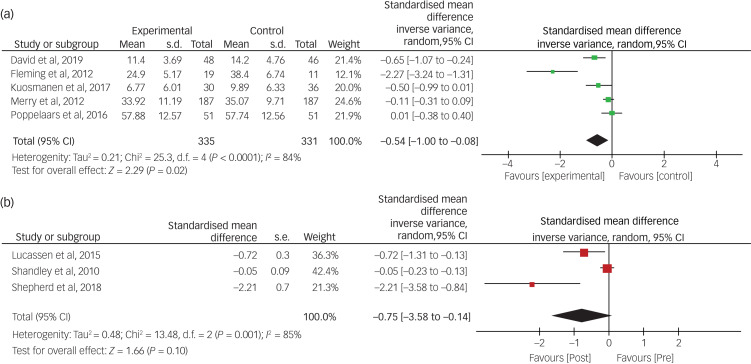
Effects of gaming interventions on youth depression for RCTs and non-RCTs. RCT, randomised controlled trial.

Figure 3 displays the effects of gaming interventions on depressive symptoms from RCTs (Fig. 3(a)) and non-RCTs (Fig. 3(b)) immediately post-intervention. For RCTs, there was a medium effect (Hedge's *g* = −0.54, 95% CI −1.00 to −0.08) favouring the experimental condition, which was statistically significant (*Z* = 2.29, *P* = 0.02). For non-RCTs, using a repeated measures design, the overall effect was relatively large (*g* = −0.75, 95% CI −1.64 to 0.14) favouring the experimental condition, but this effect was not statistically significant (*Z* = 1.66, *P* = 0.10). Heterogeneity was very high for both RCTs (*I^2^* = 84%) and non-RCTs (*I^2^* = 85%).

### Effects of gaming interventions on anxiety

**Fig. 4:**
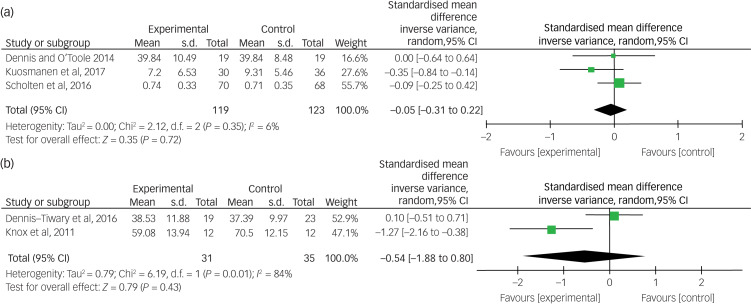
Effects of gaming interventions on youth anxiety for RCTs and non-RCTs. RCT, randomised controlled trial.

Figure 4 summarises the effects of gaming interventions on anxiety symptoms from RCTs (Fig. 4(a)) and non-RCTs (Fig. 4(b)) immediately post-intervention. For RCTs, there was minimal effect (Hedge's *g* = −0.05, 95% CI −0.31 to 0.22) favouring the experimental condition, which was not statistically significant (*Z* = 0.35, *P* = 0.72). For the two non-RCTs that used a between-participant design, the overall effect was in the medium range (*g* = −0.54, 95% CI −1.88 to 0.80) favouring the experimental condition, but was not statistically significant (*Z* = 0.79, *P* = 0.43). Heterogeneity was very low for RCTs (*I^2^* = 6%), but very high for non-RCTs (*I^2^* = 84%).

### Secondary outcomes

From all of the predefined secondary outcomes that were of interest, only Kuosmanen et al^42^ included a measure for general mental well-being (the Warwick–Edinburgh Mental Wellbeing Scale). Data extracted from this scale can be found in Table 1. Although Poppelaars et al^44^ also included a measure for suicidal ideation (the suicidal ideation subscale from the Children's Depression Inventory), this was used simply as a tool for the monitoring and management of suicide risk during the intervention, and no numerical data were reported. As such, no analysis could be carried out on any of the secondary outcomes.

## Discussion

The pooled results from both RCTs and non-RCTs show that gaming interventions designed for the treatment of mental health problems are effective for treating depressive symptoms in young people. The standardised effect sizes (Hedge's *g*) are medium to large, favouring the experimental condition regardless of study design, and are strongly manifest at the end of the intervention periods. In real-world scenarios, for example, a clinically meaningful response is usually defined as a 30% (or more) decrease in CDRS-R score.^24^ This means, assuming a normal distribution, a medium effect size of *g* = 0.5 would roughly approximate 0.5 of an s.d. (when interpreted in the same way as Cohen's *d*), which translates to around 20% change. It must be said that this is only a very primitive estimate; it is hardly the case in practice that scores from such scales conform to a normal distribution. Also, the factor of symptom severity and chronicity is not usually considered in these studies, which may have influenced treatment response (i.e. response to gaming interventions is attenuated in the more severe and chronic cases of depression). In contrast, the effectiveness of gaming interventions on anxiety symptoms in young people seems minimal from these results.

Overall, the quality of studies is moderate to good, especially with the RCTs, which suffered only minor issues with risk of bias. These biases were exclusively attrition biases where missing data at follow-up could have affected the results and potentially their interpretation. Non-RCTs were deemed not as robust as RCTs, and none of them possessed the same level of quality, as demonstrated by the ROBINS-I tool.

To our knowledge, this is the first meta-analysis focusing exclusively on the effectiveness of using serious games as an intervention in youth depression and anxiety. A previous meta-analysis investigated the effectiveness of ‘computerised therapies’ for depression and anxiety in children and young people.^51^ The authors found particularly pronounced beneficial effects of CCBT in youth (12–25 years of age) with clinically diagnosable depression (SMD −0.62, 95% CI −1.13 to −0.11) and anxiety (SMD −0.77, 95% CI −1.45 to −0.09), compared with those without clinically significant depression or anxiety. Minimal effect was found in younger children (5–11 years of age) with symptoms of depression and/or anxiety. Other computerised therapies, such as problem-solving therapy and those designed for specific phobias, did not show any significant beneficial effect; however, these did not include any gaming interventions. Although direct comparisons cannot be drawn between our results and those of Pennant et al,^51^ their findings seem to point toward higher effectiveness of CCBT in treating anxiety symptoms, whereas we found little benefit of gaming interventions for anxiety. A more recent study^52^ described the components of a new smartphone-based intervention of ‘gamified CBT’ for emotional health in young people in a naturalistic setting, yet its effectiveness has not yet been systematically evaluated.

### Limitations

Several limitations and caveats exist with this meta-analysis. First, the number of included studies is small and of those included, all had relatively small sample sizes, with only a couple of exceptions having a sample of >100 participants.^24,50^ The handful of studies with larger sample sizes reported much smaller effect sizes, which may render the pooled effect sizes less interpretable. Also, it must be noted that only two non-RCTs used a between-participant design and, as such, any estimates of heterogeneity from such a small number of studies is unlikely to be meaningful. Second, none of the studies had credibility or expectations rating pre-intervention despite non-significant baseline differences. Credibility and expectations ratings are useful when assessing potential placebo effects, as sometimes the act or even the anticipation of playing a relaxing game alone can alleviate some milder depressive and/or anxiety symptoms. Third, some studies had multiple control conditions, mainly other games or therapies of various kinds. However, to standardise between studies, we did not include any active control as a comparison. Instead, we focused on waiting lists or care as usual as control conditions, which could have inflated the effect sizes as not receiving the intervention is arguably the least effective comparison. Fourth, all studies suffered from irregular and limited follow-up time points, hence no conclusion can be drawn about the sustainability of effects from gaming interventions. This inconsistency in follow-ups is particularly pronounced in RCTs, where one study^44^ had a maximum follow-up period of 12 months and the rest had follow-up periods of no longer than 12 weeks. Finally, all studies suffered from mixed and inconsistent usage of psychometric scales or clinical assessments; some were clinician- or observer-rated and some were self-report measures, without reporting the level of convergence between observer-rated and self-report questionnaires or controlling for the divergence between them. An associated issue is that because of the variability in scales, there was no consistently defined threshold for treatment response across studies. In addition, not all studies reported the interrater reliability of assessments, and for some studies, only one assessor evaluated treatment response throughout the intervention process.

### Implications and recommendations

The findings of this meta-analysis contribute to the understanding of the effectiveness of serious games in the treatment of depressive symptoms in young people. The interactive and social component of gaming for therapeutic use may be one key factor in their effectiveness, although this was not directly assessed in our study. We know that three in five adolescents use in-game chat features and interact within gaming communities during team, multiplayer or social network games, and/or with gaming influencers/YouTubers.^11^ Gaming interventions therefore may possess a specific kind of affinity for young people, making them more acceptable and engaging compared with traditional therapeutic interventions, including those delivered via technology, such as CCBT. This is supported by a recent systematic review on broadly defined digital mental health interventions, including games, in youth depression and anxiety,^53^ which found that only highly interactive game-based activities were successful where human interaction is limited (in contrast to supervised environments), suggesting that these features are indeed appealing to young people. The authors also found that digital interventions, when pooled as a whole, were no more effective than other means of intervention otherwise.

Although we are unable to draw definitive conclusions, gaming interventions for depression in young people is a promising avenue that has the potential to improve treatment engagement. We know that young people often find purely educational materials related to therapy off-putting and boring – even when such materials are delivered digitally.^53,54^ However, the adoption and implementation of gaming interventions for youth depression in a health service context requires further investigation.

Our findings also lead to several important recommendations for future research. There is a pressing need to understand the factors underlying the effectiveness of gaming interventions, including user-friendliness, how well the graphical interface is designed, degree of interaction and levels of immersion in the gaming world. Qualitative studies would be particularly useful in eliciting user feedback, specifically as a core component of process evaluation. We need to actively incorporate young people's views of engaging with gaming interventions for treatment to enhance their feasibility and acceptability.

Also, more rigorously designed RCTs with longer-term and more regular follow-ups are also warranted. Arguably, the issue with attrition bias may worsen with longer follow-ups; hence we need much larger-scale RCTs to offset loss to follow-up. Finally, future research should assess whether gaming interventions should be used in conjunction with, or instead of, traditional therapies. Would adjunct gaming interventions consolidate the benefits of counselling or traditional CBT, for example, or would it render the latter interventions redundant? Also, future studies need to further evaluate the effectiveness of gaming interventions in youth anxiety disorders in particular, as currently there is only a small handful of studies available that investigated anxiety symptoms alone or alongside depressive symptoms, the latter of which seem to be the main target of intervention.

We were unable to evaluate the effects of using gaming interventions on other outcomes, such as quality of life and more severe symptoms including suicidal ideation, because of the extremely small number of studies with such data available. Future studies could also look at the effectiveness of gaming interventions in improving quality of life in general, contrasted with specific depression or anxiety symptoms.

Overall, by understanding the mechanisms behind responsible and effective gaming, important insights can be gained which could lead to changes in clinical practice or even youth mental health policies, such as the development of safe gaming guidelines. To the best of our knowledge, this is the first meta-analysis on the effectiveness of gaming intervention for treating depression and anxiety in youth. There is preliminary evidence to suggest that gaming interventions are an effective treatment option for youth depression, but not anxiety. Gaming intervention is an exciting emerging field with great potential, yet more high-quality studies in a variety of settings are clearly warranted to fully establish its utility, acceptability and effectiveness in treating mental health problems in young people.

## Data Availability

The manuscript reports meta-analytic data based on original studies. Extracted data are available from the corresponding author, C.H., upon request.

## Supplementary material

For supplementary material accompanying this paper visit http://doi.org/10.1192/bjo.2021.1078. click here to view supplementary material
