# Supplementary material for: The effectiveness of gaming interventions for depression and anxiety in young people: systematic review and meta-analysis
Source: BJPsych Open. 2022 Jan 7;8(1):e25. doi: 10.1192/bjo.2021.1078 (PMC8811791; doi:10.1192/bjo.2021.1078)

**Supplementary Materials**

**PRISMA-P (Preferred Reporting Items for Systematic review and Meta-Analysis Protocols) 2015 checklist: recommended items to address in a systematic review protocol***

| Section and topic | Item No | Checklist item | Page |
| --- | --- | --- | --- |
| ADMINISTRATIVE INFORMATION | | |  |
| Title: |  |  |  |
| Identification | 1a | Identify the report as a protocol of a systematic review | 1 |
| Update | 1b | If the protocol is for an update of a previous systematic review, identify as such | 1 |
| Registration | 2 | If registered, provide the name of the registry (such as PROSPERO) and registration number | 5 |
| Authors: |  |  |  |
| Contact | 3a | Provide name, institutional affiliation, e-mail address of all protocol authors; provide physical mailing address of corresponding author | 1 |
| Contributions | 3b | Describe contributions of protocol authors and identify the guarantor of the review | N/A |
| Amendments | 4 | If the protocol represents an amendment of a previously completed or published protocol, identify as such and list changes; otherwise, state plan for documenting important protocol amendments |  |
| Support: |  |  |  |
| Sources | 5a | Indicate sources of financial or other support for the review | 16 |
| Sponsor | 5b | Provide name for the review funder and/or sponsor | 16 |
| Role of sponsor or funder | 5c | Describe roles of funder(s), sponsor(s), and/or institution(s), if any, in developing the protocol | 16 |
| INTRODUCTION | | |  |
| Rationale | 6 | Describe the rationale for the review in the context of what is already known | 4 & 5 |
| Objectives | 7 | Provide an explicit statement of the question(s) the review will address with reference to participants, interventions, comparators, and outcomes (PICO) | 5 |
| METHODS | | |  |
| Eligibility criteria | 8 | Specify the study characteristics (such as PICO, study design, setting, time frame) and report characteristics (such as years considered, language, publication status) to be used as criteria for eligibility for the review | 6 |
| Information sources | 9 | Describe all intended information sources (such as electronic databases, contact with study authors, trial registers or other grey literature sources) with planned dates of coverage | 5 |
| Search strategy | 10 | Present draft of search strategy to be used for at least one electronic database, including planned limits, such that it could be repeated | 5 |
| Study records: |  |  |  |
| Data management | 11a | Describe the mechanism(s) that will be used to manage records and data throughout the review | 6 & 7 |
| Selection process | 11b | State the process that will be used for selecting studies (such as two independent reviewers) through each phase of the review (that is, screening, eligibility and inclusion in meta-analysis) | 6 & 7 |
| Data collection process | 11c | Describe planned method of extracting data from reports (such as piloting forms, done independently, in duplicate), any processes for obtaining and confirming data from investigators | 6 & 7 |
| Data items | 12 | List and define all variables for which data will be sought (such as PICO items, funding sources), any pre-planned data assumptions and simplifications | 6 & 7 |
| Outcomes and prioritization | 13 | List and define all outcomes for which data will be sought, including prioritization of main and additional outcomes, with rationale | 8 |
| Risk of bias in individual studies | 14 | Describe anticipated methods for assessing risk of bias of individual studies, including whether this will be done at the outcome or study level, or both; state how this information will be used in data synthesis | 7 |
| Data synthesis | 15a | Describe criteria under which study data will be quantitatively synthesised | 7 |
|  | 15b | If data are appropriate for quantitative synthesis, describe planned summary measures, methods of handling data and methods of combining data from studies, including any planned exploration of consistency (such as I^2^, Kendall’s τ) | 7 |
|  | 15c | Describe any proposed additional analyses (such as sensitivity or subgroup analyses, meta-regression) | 8 |
|  | 15d | If quantitative synthesis is not appropriate, describe the type of summary planned | 8 |
| Meta-bias(es) | 16 | Specify any planned assessment of meta-bias(es) (such as publication bias across studies, selective reporting within studies) | 7 |
| Confidence in cumulative evidence | 17 | Describe how the strength of the body of evidence will be assessed (such as GRADE) |  |

*** It is strongly recommended that this checklist be read in conjunction with the PRISMA-P Explanation and Elaboration (cite when available) for important clarification on the items. Amendments to a review protocol should be tracked and dated. The copyright for PRISMA-P (including checklist) is held by the PRISMA-P Group and is distributed under a Creative Commons Attribution Licence 4.0.**

*From: Shamseer L, Moher D, Clarke M, Ghersi D, Liberati A, Petticrew M, Shekelle P, Stewart L, PRISMA-P Group. Preferred reporting items for systematic review and meta-analysis protocols (PRISMA-P) 2015: elaboration and explanation. BMJ. 2015 Jan 2;349(jan02 1):g7647.*

**Search strings**

The search terms were: Games or Game-based adj1 (treatment or therap* or intervention) or computer* or video* or serious or digital* or web or internet or online or therap* or electronic or virtual adj1 (game* or gaming) in combination with Depress* or Anxi* or Stress or Mood or “Mental health” or “Mental disorder” or Psychotherap* in combination with Child* or adolesc* or "young people" or teen* or kid* or pupils or youth or juvenile or “young adult*” or “young person” or minor*.

**List of included papers**

1. David OA, Cardoș RA, Matu S. Is RET hink therapeutic game effective in preventing emotional disorders in children and adolescents? Outcomes of a randomized clinical trial. European child & adolescent psychiatry. 2019;28(1):111-22.
2. Dennis TA, O’Toole LJ. Mental health on the go: Effects of a gamified attention-bias modification mobile application in trait-anxious adults. Clinical Psychological Science. 2014;2(5):576-90.
3. Dennis-Tiwary TA, Egan LJ, Babkirk S, Denefrio S. For whom the bell tolls: Neurocognitive individual differences in the acute stress-reduction effects of an attention bias modification game for anxiety. Behaviour research and therapy. 2016;77:105-17.
4. Fleming T, Dixon R, Frampton C, Merry S. A pragmatic randomized controlled trial of computerized CBT (SPARX) for symptoms of depression among adolescents excluded from mainstream education. Behavioural and cognitive psychotherapy. 2012;40(5):529.
5. Knox M, Lentini J, Cummings T, McGrady A, Whearty K, Sancrant L. Game-based biofeedback for paediatric anxiety and depression. Mental health in family medicine. 2011;8(3):195.
6. Kuosmanen T, Fleming T, Newell J, Barry M. A pilot evaluation of the SPARX-R gaming intervention for preventing depression and improving wellbeing among adolescents in alternative education. Internet interventions. 2017;8:40-7.
7. Lucassen MF, Merry SN, Hatcher S, Frampton CM. Rainbow SPARX: A novel approach to addressing depression in sexual minority youth. Cognitive and Behavioral Practice. 2015;22(2):203-16.
8. Merry SN, Stasiak K, Shepherd M, Frampton C, Fleming T, Lucassen MF. The effectiveness of SPARX, a computerised self help intervention for adolescents seeking help for depression: randomised controlled non-inferiority trial. Bmj. 2012;344.
9. Poppelaars M, Tak YR, Lichtwarck-Aschoff A, Engels RC, Lobel A, Merry SN, et al. A randomized controlled trial comparing two cognitive-behavioral programs for adolescent girls with subclinical depression: a school-based program (Op Volle Kracht) and a computerized program (SPARX). Behaviour research and therapy. 2016;80:33-42.
10. Shandley K, Austin D, Klein B, Kyrios M. An evaluation of ‘Reach Out Central’: an online gaming program for supporting the mental health of young people. Health Educ Res. 2010;25(4):563-74.
11. Scholten H, Malmberg M, Lobel AM, Engels RCME, Granic I. A randomized controlled trial to test the effectiveness of an immersive 3D video game for anxiety prevention among adolescents. PLoS One. 2016;11(1):e0147763-e.
12. Shepherd M, Merry S, Lambie I, Thompson A. Indigenous Adolescents' Perception of an eMental Health Program (SPARX): Exploratory Qualitative Assessment. JMIR Serious Games. 2018;6(3):e13-e.

**Publication bias**

A


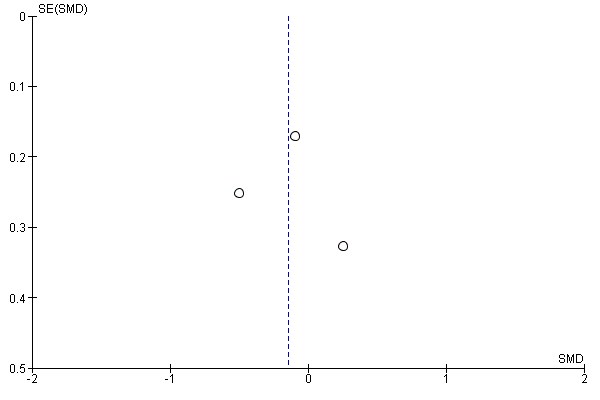


B


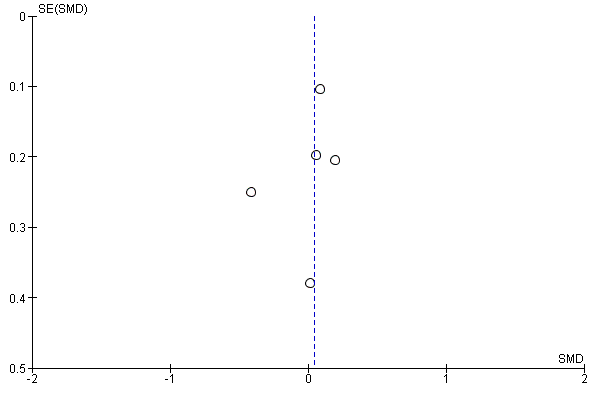


C

D


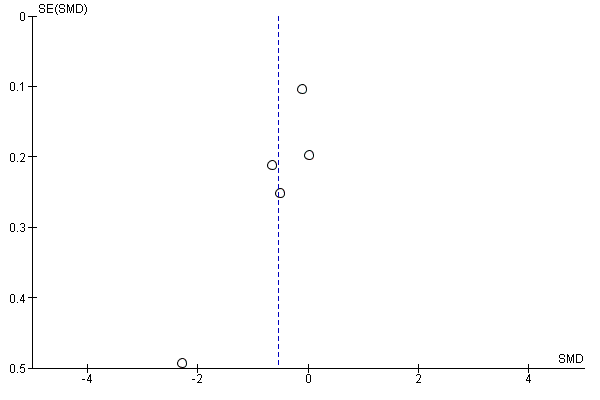

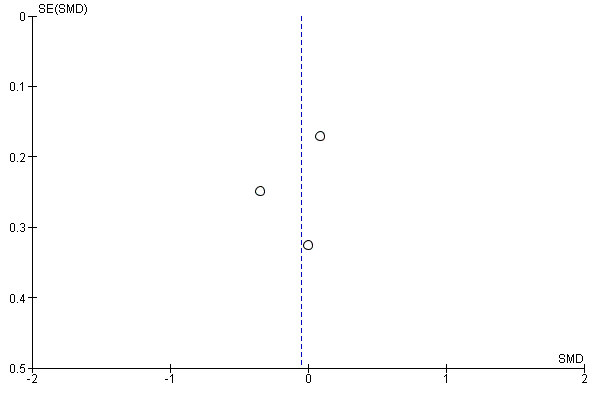


**Supplementary Figure 1.** Funnel plots showing minimal publication bias for A) Baseline comparisons for depression; B) Baseline comparisons for anxiety; C) Post-intervention comparisons for depression; and D) Post-intervention comparisons for anxiety.

**Quality assessment**


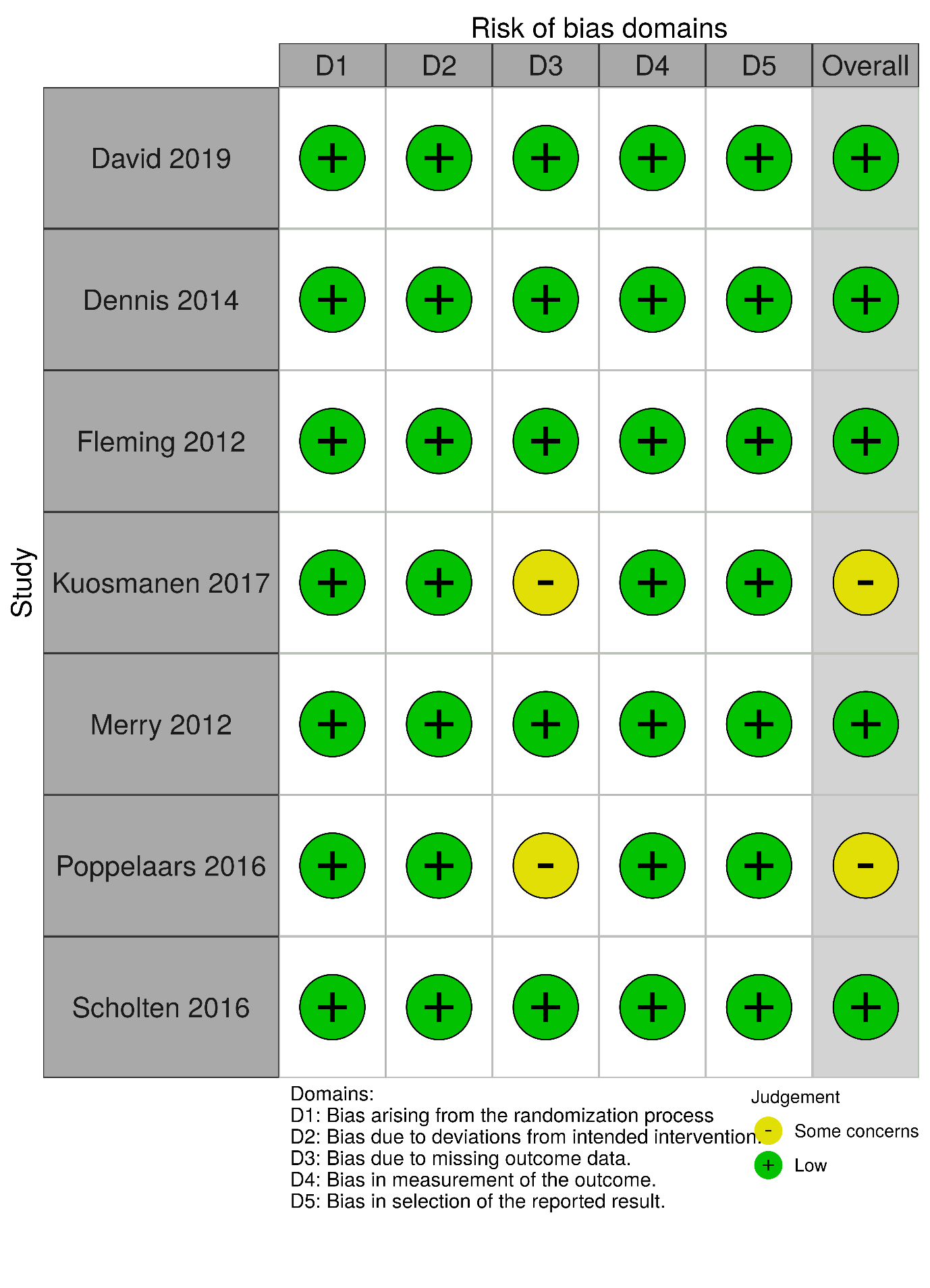


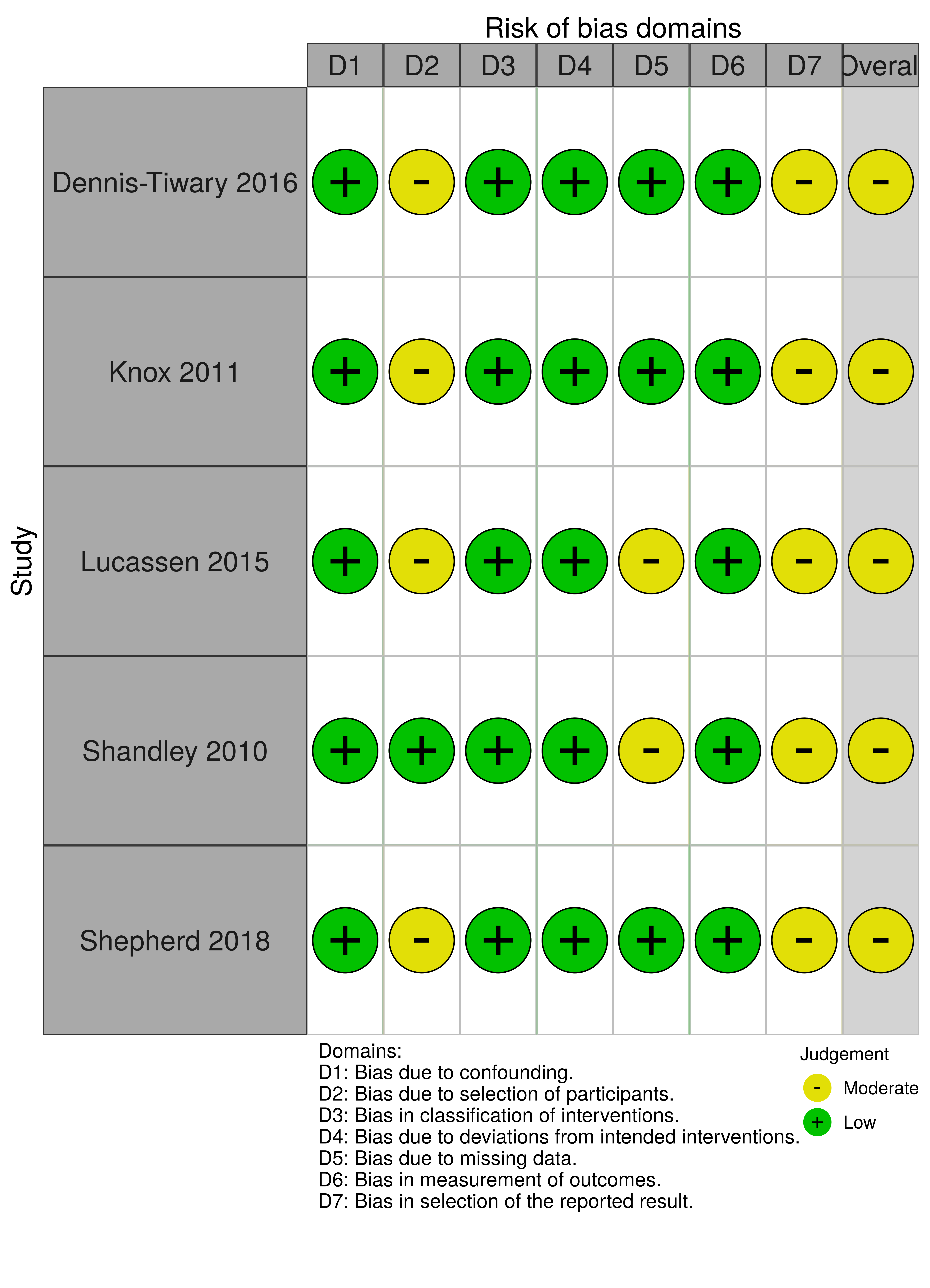


**Supplementary Figure 2**. Risk of bias assessments for randomised controlled trials (RCTs) included in the meta-analysis.


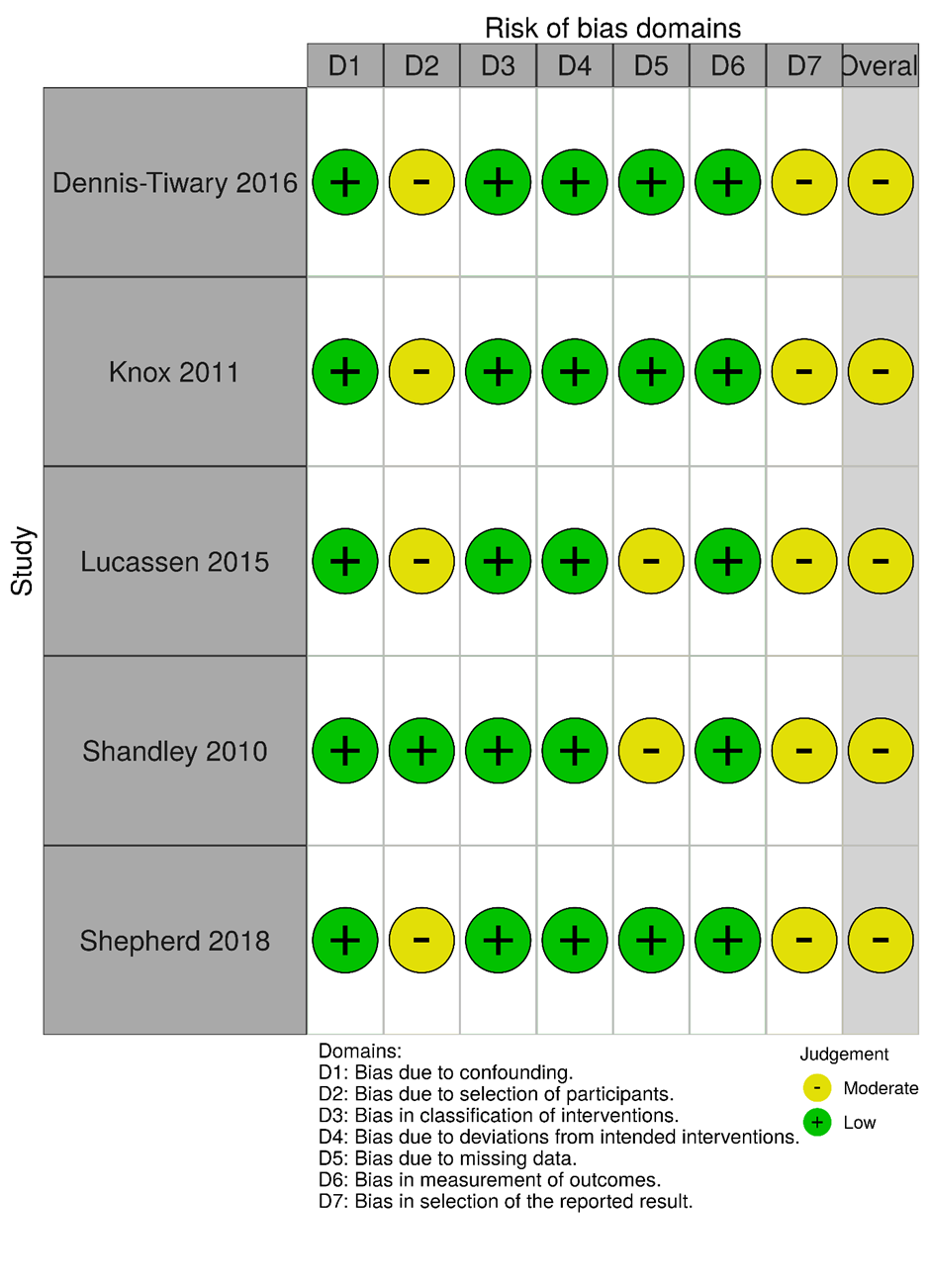


**Supplementary Figure 3**. Risk of bias assessments for non-RCTs included in the meta-analysis.

**GRADE assessments (Using GRADEPro: https://gradepro.org/)**

Baseline compared to placebo for depression and anxiety in young people

**
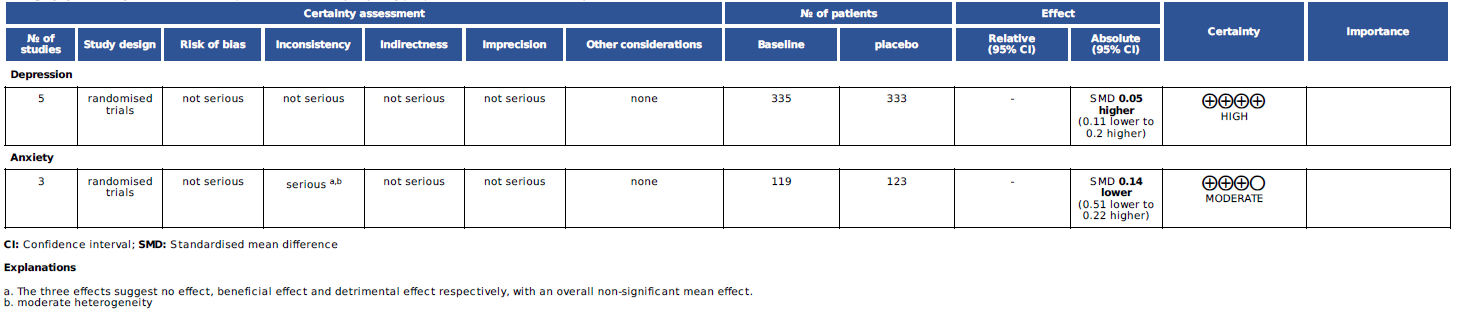
**

Post-intervention compared to placebo for depression and anxiety in young people


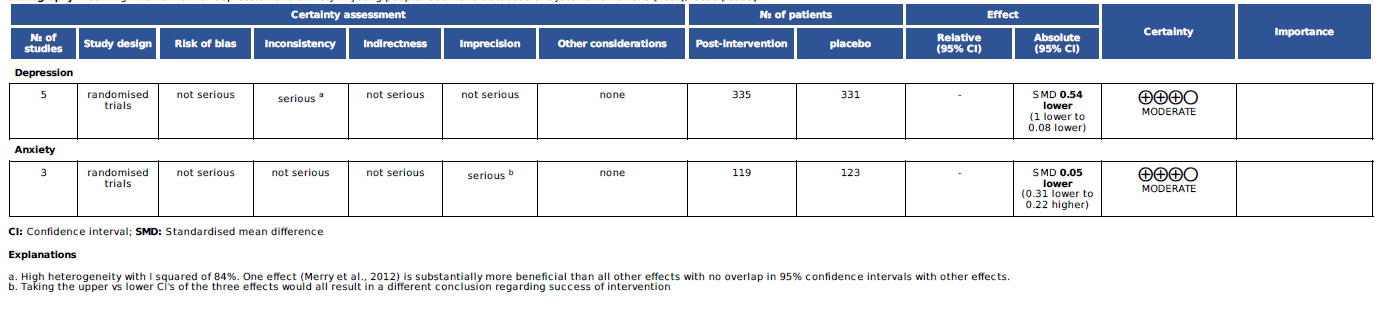

Supplement: Supplementary file 1 [file S2056472421010784sup001.doc]
